# Ethnopharmacological relevance of Chinese medicinal materials and natural products in epilepsy: a critical multi-target review integrating neurons, glia and inflammatory signaling

**DOI:** 10.3389/fphar.2026.1819195

**Published:** 2026-06-25

**Authors:** Shuolan Yan, Hongyang Li, Yao Liu, Hongni Chen, Yanxiang Zhang, Yanan Shen, Hehan Feng, Yuqian Yang, Xiuyan Wei, Lan Yuan, Simin Yang

**Affiliations:** 1 The Second School of Clinical Medicine, Southern Medical University, Guangzhou, China; 2 Department of Geriatrics, The First Hospital of Jilin University, Changchun, China; 3 Hong Kong University of Science and Technology (Guangzhou), Guangzhou, China; 4 Nanfang Hospital, Southern Medical University, Guangzhou, China; 5 Department of Pathology, West China Hospital, Sichuan University, Chengdu, China; 6 Neurosurgery Center, Zhujiang Hospital, Southern Medical University, Guangzhou, China; 7 School of Medical and Life Sciences, Chengdu University of Traditional Chinese Medicine, Chengdu, China

**Keywords:** antiseizure medications, botanical drugs, Chinese medicinal materials, epilepsy, ethnopharmacology, glia, ion channels, neuroinflammation

## Abstract

Epilepsy is a chronic neurological disorder characterized by recurrent unprovoked seizures, substantial comorbidity, and persistent pharmacoresistance in approximately one-third of affected patients. Chinese medicinal materials, including botanical drugs, selected animal-derived medicinal materials, extracts, and defined natural metabolites, have long been used as adjunctive approaches for seizure-related disorders; however, their modern pharmacological evidence remains heterogeneous and is often interpreted too broadly. This critical review synthesizes experimental, clinical, and translational evidence on 15 representative Chinese medicinal materials or natural metabolites that have been investigated in epilepsy-related models. Using a structured narrative search and an evidence-appraisal framework, we map these materials to neuronal excitability, hippocampal and entorhinal vulnerability, dentate-gyrus remodeling, microglial activation, astrocyte dysfunction, neurotransmitter balance, ion-channel regulation, and inflammatory signaling pathways including MAPK, mTOR, PI3K/AKT/FoxO1, TLR4/NF-κB, Nrf2/HO-1, and CREB. We distinguish acute seizure suppression, neuroprotection after status epilepticus, and true anti-epileptogenic or disease-modifying effects. Overall, preclinical data support multi-target biological plausibility, particularly for regulation of neuroinflammation and neuron-glia homeostasis, but most evidence remains limited by acute chemoconvulsant models, pre-treatment designs, incomplete botanical or chemical characterization, variable dose reporting, and limited high-quality clinical validation. Future studies should prioritize taxonomically validated materials, chemically characterized preparations, clinically relevant chronic seizure models, standardized outcomes, pharmacokinetic and herb-drug interaction testing, and rigorously designed randomized trials.

## 1 Introduction

Epilepsy is defined by the International League Against Epilepsy as a disease of the brain characterized by an enduring predisposition to generate epileptic seizures, with neurobiological, cognitive, psychological, and social consequences. An epileptic seizure is a transient occurrence of signs or symptoms due to abnormal excessive or synchronous neuronal activity in the brain (Fisher et al., 2014). Epilepsy remains highly prevalent in China and worldwide, and its clinical burden extends beyond recurrent seizures to cognitive decline, psychiatric comorbidity, social stigma, accidental injury, and treatment-related adverse effects, including somatic, cognitive, and psychiatric/behavioral adverse effects associated with long-term ASM exposure (Ding et al., 2021; Nevitt et al., 2022; Marson et al., 2007; Gur-Ozmen et al., 2017; Chen B. et al., 2017).

Despite major advances in antiseizure medications (ASMs), sustained seizure freedom remains difficult for many patients. Approximately one-third of individuals with epilepsy continue to have drug-resistant epilepsy even after adequate trials of appropriately selected and tolerated ASMs (Ghosh et al., 2023; Perucca et al., 2023; Ioannou et al., 2022; Gesche and Beier, 2022; Walia et al., 2004). Conventional ASMs largely target neuronal excitability through sodium channels, calcium channels, GABAergic transmission, glutamatergic signaling, or synaptic vesicle release. These mechanisms are clinically important but do not fully address the broader epileptic microenvironment, which includes chronic neuroinflammation, blood-brain barrier disruption, microglial activation, astrocyte dysfunction, aberrant synaptic remodeling, and metabolic stress (Devinsky et al., 2013; Purnell et al., 2023; Löscher et al., 2020).

Traditional Chinese medicine (TCM) has historically used botanical drugs, animal-derived medicinal materials, mineral drugs, and polyherbal formulae for seizure-related conditions. Modern pharmacological studies have increasingly examined individual botanical drugs and purified natural metabolites such as gastrodin, rhynchophylline, curcumin, wogonin, baicalin, gentiopicroside, aucubin, osthole, saikosaponin A, α-asarone, hyperoside, and scoparone (He et al., 2021; Lin and Hsieh, 2021). These studies suggest that natural products may act through convergent multi-target mechanisms, including modulation of neuroinflammation, oxidative stress, neurotransmitter cycling, ion-channel function, and neuron-glia communication. Natural alkaloids and other plant-derived metabolites have also been discussed as emerging epilepsy-relevant natural-product classes, although evidence quality varies substantially (Li et al., 2024).

However, the evidence base is heterogeneous. Many original studies use acute pentylenetetrazole (PTZ), kainic acid (KA), or pilocarpine models, with compounds administered before seizure induction. Such designs are useful for identifying antiseizure or neuroprotective signals, but they cannot by themselves prove prevention of epileptogenesis or disease modification. In addition, many reports provide limited information on botanical taxonomy, plant part, extract type, chemical characterization, dose range, route, duration, controls, and clinically meaningful outcomes. These limitations are central to interpreting the translational value of Chinese medicinal materials in epilepsy.

**FIGURE 1:**
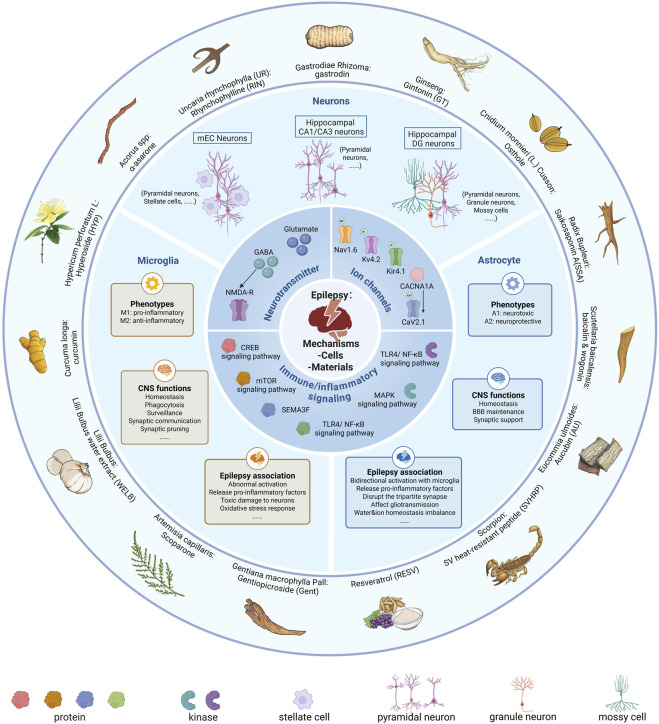
Overall target-oriented ethnopharmacological framework. The overview figure links epilepsy-relevant mechanisms, molecular targets, cell types, and representative Chinese medicinal materials or natural products. In-figure terminology has been refined to avoid overgeneralized wording: the central module now refers to Chinese medicinal materials and natural metabolites, and the inflammatory module is described as immune/inflammatory signaling. The figure is intended as a conceptual map of reviewed preclinical and translational evidence, not as proof of established clinical efficacy.

**FIGURE 2:**
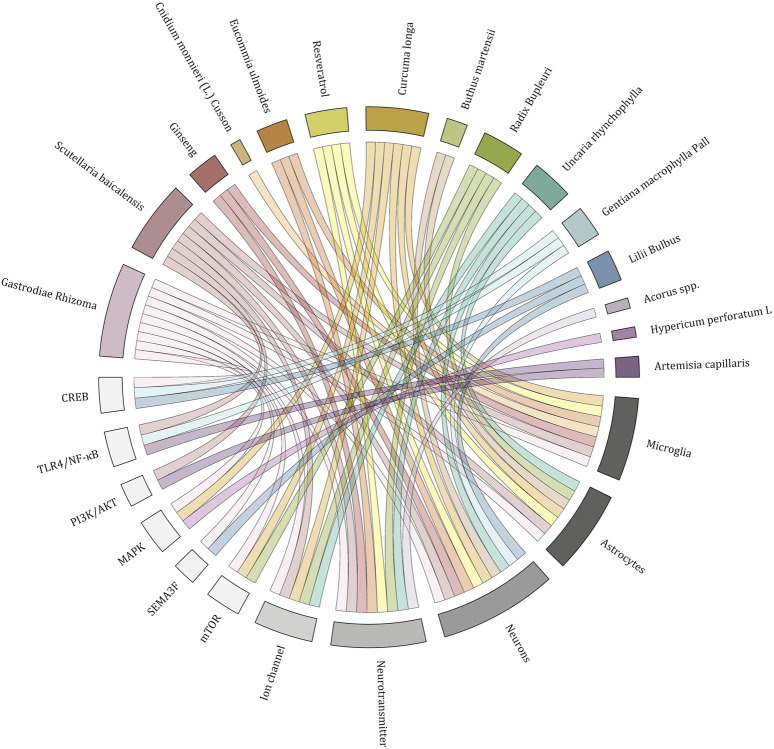
Multi-target chord network linking representative Chinese medicinal materials or natural products with epilepsy-related target modules. The colored sectors represent the reviewed materials/metabolites, and the target sectors summarize immune pathways, neurons, microglia, astrocytes, neurotransmitters, ion channels, and major signaling modules. Connecting lines indicate reported associations extracted from the reviewed literature. The network should be interpreted as an evidence-mapping and hypothesis-generating visualization rather than as a quantitative comparison of efficacy or potency.

**FIGURE 3:**
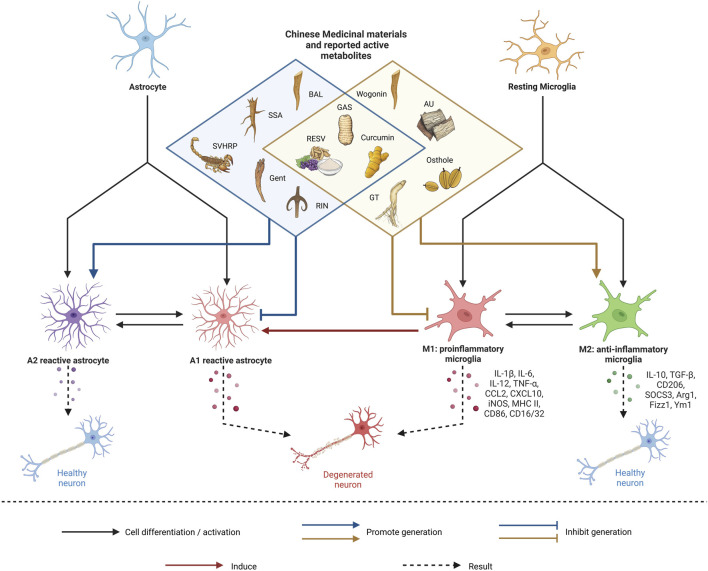
Regulation of microglial and astrocytic activation states in epilepsy models. The glial-polarization figure summarizes how seizure activity can promote pro-inflammatory microglial activation, reactive astrocytosis, cytokine release, synaptic injury, and neuronal damage. The in-figure heading has been revised to “Chinese medicinal materials and reported active metabolites” to distinguish medicinal materials, extracts, and defined metabolites more precisely. The M1/M2 and A1/A2 schemes are simplified heuristic frameworks and should not be interpreted as fixed binary cell states.

**FIGURE 4:**
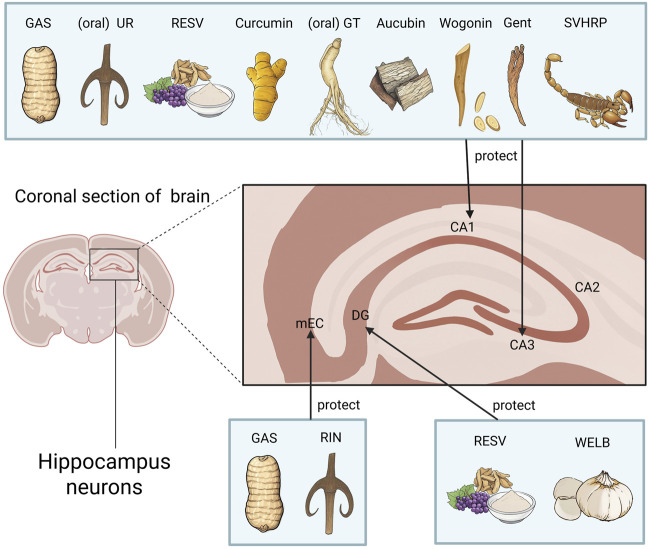
Hippocampal and entorhinal neuronal targets reported in preclinical epilepsy studies. The original hippocampal figure summarizes materials/metabolites reported to protect medial entorhinal cortex neurons, hippocampal CA1/CA3 neurons, and dentate-gyrus neurons in seizure-related models. This figure should be interpreted as a map of preclinical evidence and anatomical targeting, not as evidence of established human therapeutic efficacy.

**FIGURE 5:**
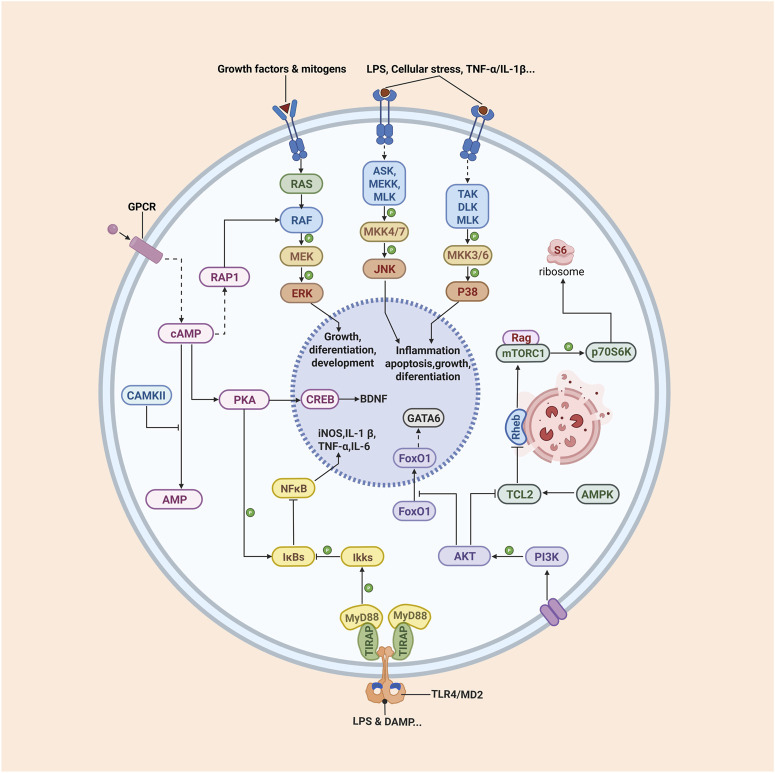
Signaling pathways implicated in reviewed epilepsy-related natural-product studies. The original signaling-pathway figure integrates MAPK, mTOR, PI3K/AKT/FoxO1, TLR4/NF-κB, and CREB-related signaling. These pathways converge on inflammatory cytokine production, oxidative stress, synaptic remodeling, apoptosis/autophagy, and neuronal excitability. The figure reflects pathway-level mechanisms reported in preclinical studies and highlights targets requiring further validation.

The novelty of this review is therefore not a simple catalogue of “effective herbs.” Instead, we provide a critical target-oriented synthesis that links Chinese medicinal materials and natural products to specific neuronal, glial, and inflammatory modules in epilepsy, while explicitly distinguishing levels of evidence, model relevance, and translational barriers. The original five figure panels are retained and aligned with the manuscript logic: Figure 1 presents the overall herb-target-cell framework; Figure 2 maps multi-target links using the circular chord network; Figure 3 summarizes glial polarization; Figure 4 highlights hippocampal and entorhinal neuronal protection; and Figure 5 integrates the key signaling pathways. A condensed evidence map is provided in Table 1, with detailed taxonomic, pharmacological, and evidence-appraisal information in Supplementary Tables S1-S4. Accordingly, this review should be interpreted as a critical evidence synthesis and hypothesis-generating framework, not as a clinical recommendation for substituting or reducing standard antiseizure medications.

**TABLE 1:** Condensed evidence map of representative Chinese medicinal materials and natural metabolites in epilepsy.

Material/Metabolite	Primary model/Evidence	Main target or cell module	Supported interpretation	Key limitation	Refs
Gastrodia elata/gastrodin	Li-pilocarpine rat; PTZ mouse; astrocyte assays	mEC neurons, Nav1.6, GABAergic transmission, MAPK/MKP-1, mTOR, astrocytes	Seizure attenuation, mEC/hippocampal neuroprotection, glial anti-inflammatory effects	Mostly acute/pre-treatment models; material and dose details vary	102,103,114,120,121
Uncaria rhynchophylla/rhynchophylline or extract	Li-pilocarpine rat; KA rat	Nav1.6/NMDA currents, CA1/CA3 neurons, astrocytes	Reduced epileptiform discharge and neuronal death	Central dosing in some mechanistic work; extract standardization limited	19,104
Resveratrol	KA rat/mouse; nanoparticle studies	DG interneurons, CA1/CA3 neurons, microglia, HMGB1/TLR4 astrocyte signaling	Neuroprotection and anti-inflammatory activity after SE	Not specific to Chinese botanical drugs; limited chronic epilepsy validation	105,106,125,126
Curcuma longa/curcumin	PTZ kindling; hippocampal-entorhinal slice culture	CA1/CA3 neurons, glia, seizure-like events	Reduced neuronal injury, glial activation, and *in vitro* epileptiform activity	Poor bioavailability; formulation-dependent effects	107,108,119
Panax ginseng/gintonin	KA mouse	LPA receptors, nrf2/HO-1, microglia/astrocytes	Seizure attenuation and hippocampal neuroprotection	Pre-treatment design; clinical epilepsy data lacking	109,116
Eucommia ulmoides/aucubin	Li-pilocarpine mouse	Autophagy, necroptosis, GABAARα1, GLT-1, HMGB1/TNF-α	Neuroprotection and neurotransmission/inflammation modulation	Dose-response and long-term seizure outcomes need confirmation	110,118
Scutellaria baicalensis/wogonin, baicalin	KA/TLE rat; Mg-free neuron-microglia co-culture; PTZ rat	Nrf2/HO-1, NF-κB, PI3K/AKT/FoxO1, astrocyte polarization	Neuroprotection, reduced microglial pruning, astrocyte modulation	Partly *in vitro*; A1/A2 astrocyte framework requires caution	111,115,122
Gentiana macrophylla/gentiopicroside	Li-pilocarpine mouse	NR2B/CaMKII/CREB, TLR4/NF-κB, astrocytes	Reduced seizures, inflammation, and hippocampal injury	High-dose pretreatment; independent replication needed	59
Buthus martensii/scorpion extract or SVHRP	KA rat; Li-pilocarpine rat; PTZ-related models	BDNF/NPY, NMDA receptors, GFAP astrocytes	Neuroprotection and astrocyte modulation	Animal-derived material; composition and safety need rigorous definition	112,123,124
Lilii bulbus/WELB	PTZ kindling mouse	Dentate gyrus, Reelin/Dab-1, c-fos, Prox1, DCX, ZnT3	Reduced DGC activation, ectopic migration, and mossy fiber sprouting	Chronic spontaneous seizure modification not established	113
Cnidium monnieri/osthole	KA-activated BV-2 cells	Notch signaling, TNF-α, IL-6, iNOS	Microglial anti-inflammatory activity	Cell-line evidence only; *in vivo* validation needed	117
Bupleurum-related/saikosaponin A	PTZ-stimulated astrocytes	AP-1/miR-155/GLAST	Regulation of astrocytic glutamate uptake	*In vitro* astrocyte model; no seizure behavior	127
Acorus-related/α-asarone	Hippocampal neurons; mechanistic models	GABAA receptors, GAD65, sodium channels	Direct modulation of inhibitory/excitability pathways	Concentration relevance and safety require caution	128–130
Hyperoside	KA mouse	MAPK, autophagy, antioxidant systems	CA3 neuroprotection	Purified metabolite; source and translation need clarification	131
Artemisia capillaris/scoparone	Pilocarpine mouse	PI3K/AKT/GSK-3β and TLR4/NF-κB	Anti-inflammatory and glia-modulatory seizure attenuation	Acute model; scoparone/scopoletin terminology must be distinct	132–134

Detailed dose, route, duration, material definition, controls, endpoints, and evidence limitations are provided in Supplementary Tables S1-S4. The table summarizes biological plausibility and does not imply that clinical disease-modifying efficacy has been established.

## 2 Review approach, search strategy, and evidence appraisal

### 2.1 Scope and objective

This article is a structured narrative and critical ethnopharmacological review rather than a quantitative systematic review or meta-analysis. The objective was to identify representative Chinese medicinal materials, botanical drugs, and natural metabolites with direct epilepsy-related evidence and to evaluate how strongly those studies support specific mechanisms across neurons, microglia, astrocytes, neurotransmitter systems, ion channels, and inflammatory signaling. Clinical studies and meta-analyses were included only to contextualize translational relevance; they were not pooled because of heterogeneity in interventions, diagnostic criteria, outcome definitions, treatment duration, and reporting quality.

### 2.2 Search strategy and study selection

We searched PubMed/MEDLINE, Web of Science, Embase, CNKI, Wanfang Data, and Google Scholar for English and Chinese publications up to May 2026. Searches combined epilepsy terms with ethnopharmacology and material-specific terms. The core search string was (“epilepsy” OR “seizure” OR “status epilepticus” OR “temporal lobe epilepsy” OR “pentylenetetrazole” OR “kainic acid” OR “pilocarpine”) AND (“Chinese herbal medicine” OR “traditional Chinese medicine” OR “botanical drug” OR “medicinal plant” OR “ethnopharmacology” OR “natural product” OR “plant metabolite”) AND (“microglia” OR “astrocyte” OR “neuroinflammation” OR “GABA” OR “glutamate” OR “ion channel” OR “MAPK” OR “mTOR” OR “PI3K” OR “NF-κB” OR “CREB” OR “hippocampus” OR “dentate gyrus” OR “entorhinal cortex”). Additional material-specific terms included Gastrodia elata, gastrodin, Uncaria rhynchophylla, rhynchophylline, resveratrol, curcumin, Panax ginseng, gintonin, aucubin, Scutellaria baicalensis, baicalin, wogonin, gentiopicroside, Buthus martensii, Lilii Bulbus, osthole, saikosaponin A, α-asarone, hyperoside, scoparone, and related Chinese drug names.

We included original *in vivo*, *in vitro*, *ex vivo*, and clinical studies if they directly investigated epilepsy, seizure models, epileptiform activity, status epilepticus, or epilepsy-related neuronal/glial outcomes. We also considered systematic reviews or meta-analyses for clinical context and network-pharmacology studies only as hypothesis-generating evidence. Studies were excluded from the mechanistic evidence table if they focused on unrelated neurological disorders without epilepsy-relevant endpoints, used only *in silico* docking without experimental or clinical validation, lacked identifiable material or active metabolite information, or did not report relevant seizure, neuroinflammatory, neuronal, glial, neurotransmitter, or pathway outcomes. Non-epilepsy natural-product studies, even when pharmacologically interesting, were not treated as direct epilepsy evidence unless seizure-related endpoints were reported (Bose et al., 2019).

### 2.3 Evidence categories and critical appraisal

For each included study, we extracted the material or metabolite, botanical or zoological source when applicable, experimental model, route, dose or concentration, treatment timing, duration, controls when reported, seizure endpoint, mechanistic endpoint, and main limitation. Evidence was categorized as clinical evidence, *in vivo* preclinical evidence, *in vitro*/*ex vivo* mechanistic evidence, or computational/hypothesis-generating evidence. We also classified the biological interpretation as seizure suppression, neuroprotection, anti-inflammatory/glia-modulatory activity, neurotransmission or ion-channel modulation, or potential anti-epileptogenic relevance. This distinction is essential because many acute seizure models demonstrate reduced seizure severity or neuronal injury but do not prove prevention of epilepsy development.

The appraisal followed the principles of best practice in ethnopharmacology and the ConPhyMP framework for medicinal plant extracts (Heinrich et al., 2022). Particular attention was given to whether studies reported taxonomically valid material names, plant part or animal source, extract or purified metabolite type, chemical characterization, dose-response information, positive and negative controls, model relevance, and clinically interpretable outcomes. Where details were absent from the reviewed literature or unavailable in the original reports, this was treated as a limitation rather than inferred.

### 2.4 Terminology and material definition

Throughout this review, “botanical drug” refers to a medicinal plant or plant part used as a medicinal material; “extract” refers to a chemically complex preparation obtained from a botanical or zoological material; and “metabolite” or “natural product” refers to a defined compound produced by plants or other natural sources. We avoid using “ingredient,” “component,” or “constituent” when referring to a defined plant metabolite. The term “Chinese herbal medicine” is retained only when discussing the broader clinical tradition or published clinical literature that uses that terminology. Full taxonomic names, authorities, families, and plant parts are provided in Supplementary Tables S2.

## 3 Pathophysiological framework for target-oriented analysis

### 3.1 Excitatory-inhibitory imbalance and neuronal hyperexcitability

Epileptic seizures arise from excessive and hypersynchronous neuronal activity. Excitatory-inhibitory imbalance remains a core concept, but it is implemented through multiple interacting processes rather than a single defect. Glutamate, GABA, and glutamine circulate between neurons and astrocytes through the glutamate-glutamine-GABA cycle (Héja et al., 2009; Sepkuty et al., 2002; Bridges and Esslinger, 2005; Albrecht and Zielińska, 2017; Schousboe et al., 2013; Kitchigina and Shubina, 2023; Bradford, 1995). Astrocytic glutamate transporters, glutamine synthetase, GAD65/GAD67, and GABAA/GABAB receptor systems are all relevant to seizure susceptibility (Treiman, 2001; Clark et al., 1992; Borden et al., 1992). Excessive glutamatergic signaling, including NMDA receptor overactivation and NR2B-related mechanisms, can amplify depolarization, excitotoxicity, and epileptiform discharge (Shao et al., 2016; Di Maio et al., 2013; Di Maio et al., 2011; Monyer et al., 1994).

Voltage-gated and inwardly rectifying ion channels further shape neuronal and glial excitability. Nav1.6 contributes to persistent and resurgent sodium currents that promote repetitive firing, whereas Cav2.1 affects presynaptic calcium-dependent transmitter release (Goldin et al., 2000; Hargus et al., 2013; Chen et al., 2011; Yue et al., 2005; Epi4K Consortium, 2016; Jouvenceau et al., 2001; Damaj et al., 2015; Imbrici et al., 2004; Ducros et al., 2001; Ophoff et al., 1996; Le Roux et al., 2021; Miao et al., 2020). Potassium channels such as Kv4.2 and astrocytic Kir4.1 help stabilize membrane excitability and extracellular potassium buffering (Köhler et al., 1996; Barnwell et al., 2009; Sun et al., 2011; Singh et al., 2006; Shibata et al., 2003; Gupte et al., 2016; Somjen, 2002; Kofuji and Newman, 2004; Ohno, 2018; Akyuz et al., 2022). Natural metabolites that modulate these systems may reduce seizure severity in preclinical settings, but ion-channel effects must be interpreted in the context of dose, route, tissue exposure, and model relevance.

### 3.2 Inflammatory and survival signaling as convergent modules

Several signaling pathways recur across epilepsy and natural-product studies. mTOR signaling influences cell growth, receptor expression, synaptic plasticity, and epileptogenic remodeling (Gerasimenko et al., 2023). SEMA3F/NPN2-related mechanisms regulate interneuron integrity, mossy fiber sprouting, and circuit remodeling. (Gant et al., 2009; Li Y. et al., 2022) MAPK signaling, including ERK, p38, and JNK branches, contributes to excitability, inflammatory cytokine production, apoptosis, and stress responses (Nateri et al., 2007; Kim and Choi, 2015; Huang et al., 2017). PI3K/AKT/FoxO1 signaling intersects with microglial motility, redox regulation, NF-κB activity, and mTOR activation (Tarassishin et al., 2011; Dong et al., 2014; Vieira et al., 2021; Henshall et al., 2002; Wang et al., 2019; Seong et al., 2023). TLR4/NF-κB signaling drives the expression of IL-1β, TNF-α, IL-6, iNOS, COX-2, and other mediators that can lower seizure threshold and impair inhibitory control (Huang et al., 2024; Li et al., 2011; Tian et al., 2024; Wang et al., 2021). CREB signaling has context-dependent roles in synaptic plasticity, neuroprotection, and excitability (Tian et al., 2024; Huang et al., 2025; Kim et al., 2024).

### 3.3 Neuronal and glial cellular substrates

Hippocampal and entorhinal circuits are central to temporal lobe epilepsy. The entorhinal cortex, dentate gyrus, CA3, and CA1 form a network in which selective neuronal vulnerability, aberrant neurogenesis, interneuron loss, mossy fiber sprouting, and impaired dentate gating can support seizure propagation (Knierim, 2015; Bartsch et al., 2015; Einenkel and Salameh, 2024; Borzello et al., 2023; Li et al., 2013; Krook-Magnuson et al., 2015; Schwarcz et al., 2000). Microglia and astrocytes are not passive bystanders. Microglia can adopt pro-inflammatory or reparative activation states, releasing cytokines and modulating synaptic pruning (Yu et al., 2023; Liu et al., 2011; Devinsky et al., 2013; Orihuela et al., 2016; Boche et al., 2013; Tang and Le, 2016). Astrocytes regulate glutamate clearance, potassium buffering, water balance, blood-brain barrier function, gliotransmission, and inflammatory amplification (Purnell et al., 2023; Díaz et al., 2023; Hsu et al., 2007; Vezzani et al., 2022). Thus, neuron-glia interactions provide a biologically plausible framework for multi-target ethnopharmacological interventions.

### 3.4 Distinguishing seizure suppression, neuroprotection, and anti-epileptogenesis

A central interpretive issue is the distinction between acute antiseizure activity, post-seizure neuroprotection, and true anti-epileptogenic or disease-modifying effects. Reduced Racine score, longer seizure latency, lower seizure frequency in acute PTZ/KA/pilocarpine models (Hui Yin et al., 2013), or reduced neuronal loss after status epilepticus may support antiseizure or neuroprotective activity. These endpoints do not automatically demonstrate prevention of epilepsy development, especially when treatment is given before the convulsant challenge. True anti-epileptogenic evidence would require delayed treatment designs, longitudinal spontaneous seizure monitoring, validated chronic epilepsy models, and demonstration that treatment modifies the development or persistence of epileptic networks rather than only suppressing acute seizures. This review therefore uses conservative language and identifies disease-modification as a future research question rather than an established clinical conclusion.

## 4 Ethnopharmacological evidence across neuronal, glial, and inflammatory targets

### 4.1 Entorhinal and hippocampal neuronal excitability

Gastrodin from Gastrodia elata Blume [Orchidaceae; Gastrodiae Rhizoma] and rhynchophylline from Uncaria rhynchophylla (Miq.) Miq [Rubiaceae; Uncariae Ramulus Cum Uncis] have been investigated in lithium-pilocarpine models of temporal lobe epilepsy. Gastrodin reduced status epilepticus severity and protected layer III medial entorhinal cortex neurons, with electrophysiological evidence implicating Nav1.6 persistent and resurgent sodium currents (Shao et al., 2017). Rhynchophylline reduced seizure severity in pre-treatment and post-treatment paradigms and suppressed epileptiform discharges in entorhinal cortex neurons, involving persistent sodium current and NMDA receptor current inhibition (Shao et al., 2016). These studies are mechanistically valuable because they combine behavioral, histological, and electrophysiological endpoints. Their limitations include reliance on chemically induced rodent models, central or experimental dosing strategies in some protocols, and limited evidence for long-term spontaneous seizure modification.

Hippocampal CA1 and CA3 neurons are frequent targets in natural-product studies. Gastrodin injection reduced neuronal injury in CA1/CA3 after pilocarpine-induced seizures and was linked to enhanced GABAergic transmission (Yang et al., 2021). Oral Uncaria rhynchophylla extract reduced KA-induced seizures, abnormal CA1 discharges, neuronal death, glial proliferation, and S100B changes (Lin and Hsieh, 2011). Resveratrol reduced hippocampal neurodegeneration and epileptiform discharge after KA-induced status epilepticus (Mishra et al., 2015; Li et al., 2017). Curcumin and curcumin-loaded nanoparticles reduced PTZ-kindling-associated neuronal injury and glial activation, whereas hippocampal-entorhinal slice cultures suggested that curcumin may reduce seizure-like events *in vitro* (Hashemian et al., 2017; Drion et al., 2019). Gintonin from Panax ginseng C.A.Mey [Araliaceae] reduced KA-induced seizures and hippocampal neuronal death through LPA receptor, anti-inflammatory, and antioxidant mechanisms (Choi et al., 2023; Choi et al., 2018). Aucubin from Eucommia ulmoides Oliv [Eucommiaceae] attenuated lithium-pilocarpine hippocampal injury by modulating autophagy, necroptosis, neurotransmission, and neuroinflammation (Wang et al., 2017; Chen et al., 2019). Wogonin from Scutellaria baicalensis Georgi [Lamiaceae] and gentiopicroside from Gentiana macrophylla Pall [Gentianaceae] showed neuroprotective and anti-inflammatory effects in seizure models (Tian et al., 2024; Guo et al., 2020).

Taken together, these CA1/CA3 studies show consistent signals for reduced neuronal injury and inflammatory stress. However, many used pre-treatment designs or acute/subacute endpoints. They should therefore be described as supporting neuroprotection and seizure attenuation rather than proving established disease-modifying therapy.

### 4.2 Dentate-gyrus remodeling and aberrant neurogenesis

The dentate gyrus is a key gate for cortical input into hippocampal seizure circuits. Resveratrol reduced the loss of GABAergic interneuron subtypes and reelin-positive interneurons after status epilepticus and attenuated abnormal migration of DCX-positive newborn neurons (Mishra et al., 2015). Water extract of Lilii Bulbus reduced excessive dentate granule cell activation, ectopic dentate granule cells, mossy fiber sprouting, and changes in Reelin/Dab-1-related signaling in PTZ-kindled mice (Park and Cai, 2024). These findings are relevant because they address structural remodeling rather than only acute seizure behavior. Nevertheless, chronic spontaneous seizure monitoring and delayed intervention designs are still needed before classifying these effects as anti-epileptogenic.

### 4.3 Microglial activation and inflammatory signaling

Microglia-centered studies provide one of the strongest mechanistic themes in this literature. Gastrodin reduced PTZ-induced IL-1β and TNF-α while increasing IL-10 and modulating MAPK/MKP-1-related inflammatory responses (Chen L. et al., 2017). Wogonin activated PI3K/AKT/FoxO1 signaling in microglia-neuron co-cultures, reducing inflammatory cytokine secretion, microglial motility, excessive phagocytosis, and synaptic over-pruning (Hu et al., 2024). Gintonin inhibited KA-induced microglial and astrocytic activation, suppressed IL-1β, IL-6, COX-2, and iNOS, and showed mechanistic dependence on LPA receptors (Choi et al., 2023). Osthole from Cnidium monnieri (L.) Cusson [Apiaceae] inhibited KA-activated BV-2 microglial proliferation and inflammatory cytokine release through downregulation of Notch signaling (Li et al., 2020). Aucubin and resveratrol also reduced microglial inflammatory activation in seizure models (Mishra et al., 2015; Chen et al., 2019).

These studies support a coherent anti-inflammatory and glia-modulatory hypothesis. The critical limitation is that BV-2 cell studies and acute inflammatory readouts may not fully represent *in vivo* human microglial states or long-term epilepsy progression. Future work should include primary microglia or human induced pluripotent stem cell-derived glia, cell-type-specific transcriptomics, seizure-electrophysiology correlation, and chronic outcome measurement.

### 4.4 Astrocyte dysfunction, glutamate handling, and reactive states

Astrocytes maintain extracellular glutamate and potassium homeostasis and can amplify neuroinflammation after seizures. Gastrodia elata water extract reduced astrocytic proliferation and mTOR overexpression in pilocarpine mice, whereas gastrodin protected astrocytes from LPS-induced autophagy-associated cell death and apoptosis *in vitro* (Yip et al., 2020; Wang et al., 2016). Baicalin reduced the proportion of A1-like astrocytes in PTZ-induced epileptic rats and may promote a shift toward a more protective astrocytic phenotype (Li G. et al., 2022). Scorpion ethanol extract and scorpion venom heat-resistant peptide reduced GFAP-related astrocyte activation in seizure models (Liang et al., 2012; Sui et al., 2024; Chen et al., 2021). Resveratrol inhibited astrocytic HMGB1/TLR4 signaling and acute seizure-related inflammation (Siddiqui et al., 2022; Wang et al., 2004). Saikosaponin A from Bupleurum-related botanical drugs regulated astrocytic glutamate uptake through the AP-1/miR-155/GLAST pathway in PTZ-stimulated astrocytes (Gao et al., 2017). Curcumin, Uncaria rhynchophylla extract, and gentiopicroside also reduced GFAP-positive astrocyte activation in relevant models (Tian et al., 2024; Lin and Hsieh, 2011; Hashemian et al., 2017; Kaur et al., 2015).

The astrocyte evidence is mechanistically attractive because it links natural products to glutamate uptake, reactive gliosis, mTOR, HMGB1/TLR4, and inflammatory cytokines. However, astrocytic A1/A2 terminology is an oversimplified framework, and GFAP reduction alone does not establish functional restoration of astrocyte homeostasis. Studies should add glutamate transporter function, potassium buffering, calcium signaling, aquaporin-4 distribution, blood-brain barrier readouts, and seizure electrophysiology.

### 4.5 Neurotransmitter, ion-channel, and signaling-pathway mechanisms

Several defined metabolites target neurotransmission or channel-linked excitability. α-Asarone, reported from Acorus-related botanical drugs, directly activated GABAA receptor-mediated chloride currents in hippocampal neurons and interacted with GABAergic signaling; additional studies implicated GAD65, GABAA receptors, and voltage-activated sodium channels (Huang et al., 2013; Liu et al., 2015; Wang et al., 2014). Hyperoside protected against KA-induced neuronal damage through antioxidant effects, MAPK modulation, and autophagy regulation (Cao et al., 2020). Scoparone from Artemisia capillaris Thunb [Asteraceae; Artemisiae Scopariae Herba] reduced pilocarpine-induced seizures and inhibited PI3K/AKT/GSK-3β and TLR4/NF-κB inflammatory signaling (Cai et al., 2020; Jung et al., 2019; Xia et al., 2018). The terminology is clarified here: scoparone is 6,7-dimethoxycoumarin, whereas scopoletin is a different coumarin metabolite.

Network pharmacology studies have proposed broad target sets for Phyllanthus emblica L., Rhizoma Coptidis, and other botanical drugs (Li et al., 2023; Xiao et al., 2025; Huang et al., 2022; Mai et al., 2026; Sun et al., 2025). These studies are useful for hypothesis generation, especially for identifying multi-target patterns, but they should not be treated as efficacy evidence unless supported by experimental validation. Therefore, computational evidence is discussed separately from *in vivo*, *in vitro*, and clinical evidence.

### 4.6 Critical synthesis of the preclinical evidence

Across the preclinical literature, the most consistent signals are reduced seizure severity or latency changes in acute models, decreased neuronal injury in hippocampal and entorhinal structures, reduced microglial or astrocytic activation, suppression of pro-inflammatory cytokines, and modulation of MAPK, PI3K/AKT/FoxO1, TLR4/NF-κB, Nrf2/HO-1, mTOR, and neurotransmitter-related pathways. These findings support biological plausibility for multi-target interventions but also reveal several limitations: incomplete material characterization, inconsistent dose-response testing, frequent pre-treatment designs, limited positive-control reporting, scarce chronic spontaneous seizure monitoring, variable sex and age reporting, and insufficient pharmacokinetic data. Therefore, the current evidence should be interpreted as a preclinical mechanistic foundation, not as proof that these botanical drugs or metabolites are clinically established anti-epileptogenic therapies.

## 5 Clinical evidence and translational limitations

### 5.1 Clinical evidence: suggestive but heterogeneous

Clinical studies of Chinese herbal medicine for epilepsy have reported potential benefits when used as adjunctive therapy with ASMs. Systematic reviews and meta-analyses have suggested improvements in seizure-related outcomes and adverse-effect profiles in some settings, including refractory epilepsy and post-stroke epilepsy. (Zhao C. et al., 2022; Luo et al., 2025; Zhao Y. et al., 2022; Sun et al., 2023; Lu et al., 2023) However, these conclusions require careful interpretation because many included trials are small, single-center, incompletely blinded, and heterogeneous in diagnostic criteria, intervention composition, ASM background therapy, follow-up duration, and outcome definitions.

A particularly important issue is the “total effective rate,” which is commonly reported in Chinese clinical studies but is not a standardized international epilepsy outcome. Its definition varies across studies and may combine seizure-frequency reduction, symptom improvement, clinician judgment, and sometimes electroencephalographic changes. Here, total effective rate is defined as a non-standard composite outcome when reported, and it is not treated as equivalent to seizure freedom or internationally accepted responder outcomes. More clinically interpretable outcomes include seizure-frequency reduction, ≥50% responder rate, seizure-free rate, time to seizure recurrence, ASM dose reduction, adverse events, quality of life, cognitive outcomes, and long-term relapse.

Evidence for specific purified metabolites in human epilepsy is still limited. Curcumin nanomicelles have been explored in pediatric intractable epilepsy, and cannabidiol studies provide a broader phytochemical comparator, although phytocannabinoids are not the main focus of Chinese medicinal-material evidence (Erfani et al., 2022; Hill et al., 2012; Huntsman et al., 2019; Reithmeier et al., 2018; Gaston and Szaflarski, 2018). Pharmacokinetic work suggests that Paeoniae Radix may not substantially alter valproic acid pharmacokinetics in healthy volunteers, but this does not prove clinical antiseizure efficacy (Chen et al., 2000). Overall, clinical evidence should be described as preliminary and hypothesis-supporting rather than confirmatory.

### 5.2 Pharmacokinetic, safety, and formulation barriers

Several translational barriers are shared across these materials. First, many metabolites have poor solubility, limited blood-brain barrier penetration, or unclear brain exposure. Curcumin is a clear example, where nanoparticle and nanomicelle formulations may improve solubility and bioavailability but also alter pharmacokinetics and safety (ERFANI et al., 2022; Hashemian et al., 2017). Second, extract composition may vary by species, plant part, geography, processing, extraction solvent, and batch. Third, herb-drug interactions are highly relevant because most patients will continue ASMs. Fourth, safety evidence for long-term adjunctive use is often incomplete, especially in children, pregnant patients, elderly patients, and individuals with hepatic or renal impairment.

### 5.3 Quality control and taxonomic requirements

Ethnopharmacological evidence requires precise material definition. Each botanical drug should be reported with a valid Latin binomial, authority, family, plant part, voucher or authentication method, processing method, extract solvent, extraction ratio, chemical fingerprint, marker metabolite content, and storage conditions. Polyherbal formulae should include the complete composition and proportions of all botanical and non-botanical materials. In this synthesis, polyherbal preparations such as Dingxian Pills and Chaihu Guizhi Decoction are mentioned only as historical or clinical-context examples unless sufficient composition and mechanistic data are available. The primary mechanistic synthesis focuses on individual materials or defined metabolites with direct epilepsy-related data.

## 6 Future research priorities

Future research should move from descriptive target lists to reproducible, model-appropriate, and clinically meaningful validation. Priority areas include: (1) taxonomic validation of all botanical materials using recognized nomenclatural resources and documentation of voucher specimens; (2) chemical characterization of extracts using chromatographic or metabolomic profiling, marker-content quantification, and batch-to-batch quality control; (3) dose-response and pharmacokinetic studies that measure systemic and brain exposure; (4) use of clinically relevant chronic epilepsy models, delayed-treatment designs, and long-term spontaneous seizure monitoring; (5) separation of acute seizure suppression, neuroprotection, and anti-epileptogenic endpoints; (6) incorporation of positive and negative controls, randomization, blinding, and transparent reporting; (7) cell-type-specific validation of microglial and astrocytic mechanisms using primary cells, human-derived systems, or single-cell omics; (8) assessment of herb-ASM interactions and long-term safety; and (9) multicenter randomized trials using internationally accepted epilepsy outcomes.

Multi-omics, network pharmacology, and systems pharmacology can generate hypotheses about multi-target actions, but these approaches should be integrated with experimental validation rather than used as standalone evidence of efficacy. A cautious translational route may be an adjunctive strategy in which chemically characterized botanical preparations or defined metabolites are tested alongside standard ASMs, with careful monitoring of efficacy, adverse events, and pharmacokinetic interactions.

## 7 Conclusion

Chinese medicinal materials and natural products offer a biologically plausible multi-target framework for epilepsy research because they can influence neuronal excitability, hippocampal injury, dentate-gyrus remodeling, microglial activation, astrocyte dysfunction, neurotransmitter balance, ion-channel regulation, oxidative stress, and inflammatory signaling. The strongest current evidence is preclinical and supports seizure attenuation, neuroprotection, and neuron-glia modulation in PTZ, KA, pilocarpine, and related models. However, these data should not be overinterpreted as proof of clinical disease modification. A rigorous translational pathway will require taxonomically validated materials, chemically characterized preparations, standardized dosing, pharmacokinetic and safety assessment, chronic epilepsy models, and high-quality clinical trials with accepted seizure and patient-centered outcomes. With these improvements, ethnopharmacological research may generate testable adjunctive strategies for refractory epilepsy while meeting modern standards of reproducibility and evidence-based medicine.
